# A qualitative study of an integrated maternity, drugs and social care service for drug-using women

**DOI:** 10.1186/1471-2393-6-19

**Published:** 2006-06-13

**Authors:** Jennifer L Hall, Edwin R van Teijlingen

**Affiliations:** 1 Public Health Lead, Aberdeenshire CHP, Inverurie Hospital, Inverurie, UK; 2 Department of Public Health & Dugald Baird Centre for Research on Women's Health, University of Aberdeen, Aberdeen, UK

## Abstract

**Background:**

The care of drug-using pregnant women is a growing health and social care concern in many countries. A specialist clinic was established offering multidisciplinary care and advice to pregnant drug users in and around Aberdeen (UK) in 1997. The majority of women stabilise and reduce their drug use. By determining the needs and views of the women more appropriate services and prevention strategies may be developed. There has been little research conducted in this area and none in Scotland.

**Methods:**

This is a qualitative study that aimed to gain an understanding of the experiences of women drug users, seeking and receiving prenatal care and drug services from a specialist clinic. Twelve women participated in semi-structured one-to-one interviews.

**Results:**

The women preferred the multidisciplinary clinic (one-stop shop) to traditional prenatal care centred within General Practice. The relationships of the clients to the range of Clinic professionals and in hospital were explored as well as attitudes to Clinic care. The study participants attributed success in reducing their drug use to the combination of different aspects of care of the multi-agency clinic, especially the high level prenatal support. It is this arrangement of all aspects of care together that seem to produce better outcomes for mother and child than single care elements delivered separately. Some women reported that their pregnancy encouraged them to rapidly detoxify due to the guilt experienced. The most important aspects of the Clinic care were found to be non-judgemental attitude of staff, consistent staff, high level of support, reliable information and multi-agency integrated care.

**Conclusion:**

There is an impetus for women drug users to change lifestyle during pregnancy. The study highlighted a need for women to have access to reliable information on the effects of drugs on the baby.

Further research is required to determine whether positive outcomes related to clinic attendance in the prenatal period are sustained in the postnatal period. Early referral to a specialist clinic is of benefit to the women, as they reported to receive more appropriate care, especially in relation to their drug use. A greater awareness of needs of the pregnant drug user could help the design of more effective prevention strategies.

## Background

The number of new drug users in contact with services in Scotland [1] is increasing and over the last five years has risen from 9,128 to 11,472 (total population of Scotland is just over 5 million). The majority of females reported to the national database are of childbearing age. Opiates/opioids, especially, heroin, are the main illegal drugs in the UK [2], and heroin is also the main drug of pregnant drug users. Many pregnant drug users stabilise on the synthetic heroin substitute, methadone. Polydrug use is common. Other drugs commonly used by this group are (crack) cocaine, amphetamines, benzodiazepines and cannabis [3].

In Scotland, the rate per 1000 discharges of maternities recording drug use has risen from 3.6 in 1998/9 to 6.7 in 2002/03 [1]. The number of babies withdrawing from opioids in Aberdeen Maternity Hospital was 1 or 2 per year in the 1980s, rising to 8 in 1995 and 63 in 2000 [3]. In May 2005 a local newspaper reported that the "number of pregnant women with drug problems giving birth at the hospital has soared ... to nearly 100" [4].

The foetal effects of opiate use include premature birth, low birth weight, incidence of sudden infant death syndrome (SIDS or cot death), small head circumference, and neonatal withdrawal symptoms [5-9]. The association of drug use with socio-economic deprivation prevents any single symptom in the foetus being attributed to drug use. The only specific effect of maternal drug use on the foetus is neonatal withdrawal, or neonatal abstinence syndrome (NAS). [8]

The stigma of drug use is traditionally greater for women than for men [10], and is even more pronounced for pregnant drug users. They may suffer anxiety and guilt over the effect of the drugs on the baby, and fear of losing the child into care by Social Work (SW) [11]. In the UK, SW have a legal obligation towards protection of children, this has led to children being taken into care against the will of the parents. This obligation has given SW a 'bad' name in certain parts of society, especially among drug users. It is stipulated that drug use alone should not be the sole reason for separating a mother from her child [12]. Women may avoid facing reality and avoid contact with all services, especially prenatal services [13]. The importance of providing appropriate services to meet the needs of pregnant drug users is acknowledged. [14]

There is evidence that drug-using women attend antenatal care late and/or conceal their drugs use to maternity care providers [6,15]. To be effective, prenatal care should be appropriate to women's needs, easily accessible and the woman should be involved in the planning of care [16,17]. A higher frequency of prenatal care and social support for this group has been associated with improved outcomes in terms of infant birth weight [18,19].

Women should not be deterred from seeking prenatal care [20]. It has been recognised that providing special services to attract and retain pregnant women drug users into prenatal care can help to address their complex problems [2]. Multidisciplinary services can address drug use at the same time as providing prenatal care [21]. A non-judgemental approach has been advocated to encourage women into services [8].

Specialised services for pregnant drug users were established in the UK from the late 1980s onwards. In Scotland, only two such services exist, in Glasgow and Aberdeen. Since 1990, the Glasgow Women's Reproductive Service has provided a service for women with any type of social problem. Half of all its clients are drug users [22].

The Golden Square Special Clinic in Aberdeen (henceforth referred to as the Aberdeen Clinic) opened in September 1997 within a family planning clinic in the city centre. The Aberdeen Clinic is community-based multi-disciplinary agency, and it also offers a greater frequency of prenatal care than available to the average pregnant woman in the area. The multi-disciplinary team consists of an obstetrician; a community midwife; a community psychiatric nurse from the statutory drugs service called the Substance Misuse Service (SMS); a social worker and a drugs worker from Drugs Action (DA), a voluntary drugs service, which provides counselling and social support. The local hospital offers detoxification and designated space is always available [3]. However, only a minority of drugs misusing pregnant women use this facility. The overwhelming majority of the clients attending Aberdeen Clinic were prescribed substitute drugs, mainly methadone to stabilise their drug use.

Identification of pregnant drug users by the services and stabilisation of their drug habit before labour can simplify their clinical management [23]. The desire to have a healthy baby can give an impetus to change drug use [15] and pregnancy may be regarded as a window of opportunity for the services as well as for the client, with possible long-term benefits [24]. This is especially relevant when child protection issues are considered [25]. A mother is more likely to retain contact with the services after the birth if a good relationship with a service has been established before the birth with positive implications for the future well-being of the child [26].

Taking account of client views is regarded as one of the core principles of effective practice in the provision of drug misuse services [27]. User-friendly services may be developed by determining women's views [28].

Much research has covered the effect of maternal drug misuse on the baby, but there has been little work exploring the experiences of pregnant drug users and attempting to understand their needs [11,15]. Hepburn and Elliott [22] showed that staff attitudes were more important to clients than medical aspects of care; in all cases pregnancy provided a strong motivation for change. Klee and Jackson [10] studied a cohort of 51 female drug users from pregnancy through childbirth and into motherhood. Most believed pregnancy was a good time to reduce their drug use, but many had reverted to street drugs or increased their methadone dosage by the end of the study. Overall, satisfaction with treatment was highest if it was an integrated service and especially if a specialist person, such as a drugs-liaison midwife, was involved.

This study explores the experiences of women drug users who have received their prenatal care from a specialised multi-agency clinic.

## Methods

A short questionnaire determining background details was administered before a semi-structured taped interview. The questionnaire was based on previous reported studies with drug users [10,15]. Administering the questionnaire before the interview allowed a relationship to develop between the researcher and the interviewee thus increasing the likelihood of responses being elicited to further qualitative questions [29]. The quantitative method was employed in this way to maximise the potential of the qualitative method [30].

### Sample

Women, who were pregnant or who had had a baby in the past three years, and who were recent or current drug users, and who had had contact with the Aberdeen Clinic were sought for a research interview (this constitutes approximately 40 potential interviewees [3]). Due to UK ethics regulations we could not approach women directly, therefore recruitment was facilitated by service providers, who offered all eligible women a leaflet about the study. The following service providers helped in the recruitment process: the Aberdeen Clinic; the local voluntary drugs service (DA); and Social Work (SW). We asked these services to approach both women currently pregnant and using the Aberdeen Clinic as well as those women who were postpartum and recently used the service. Full ethical approval was granted by the regional ethics committee (Grampian Research Ethics Committee) and confidentiality and anonymity has been maintained.

### Semi-structured interview

The interviews were conducted as informally as possible; in the interviewee's home or at the clinic or service they were attending [31]. The interview schedule (Table 1) was developed such that the order could be varied if required [32].

**Table 1:** Interview schedule

• Experiences of seeking care – route of referral to the Clinic; feelings; ease of access;
• Range of care received
• Experiences of receiving care – feelings about the care; care at the Clinic compared to other settings: positive and negative experiences;
• Relationship with health care professionals – feelings about the staff; consistency of staff; relationships with staff from other services; type of relationship; hospital relationships;
• Drug use in pregnancy – feelings about drug use during pregnancy; change of drug use; reasons for change;
• Perceptions of available service options and suggestions

### Data analysis

Interviews were tape-recorded with permission and transcribed, with field notes written directly after each interview. Key points in the transcripts were categorised using Content Analysis [33,34]. The transcripts were independently analysed by two researchers to ensure inter-rater reliability [35].

Care was taken to ensure that all selected quotations, following a particular statement of text, are from different individuals. This policy was adopted instead of noting the origin of each quotation in the text, as, due to the small size of the sample there is the potential of an individual interviewee being identified [36].

## Results

### Quantitative questionnaire

Twelve women participated who had all received care from the Aberdeen Clinic: four interviewees were pregnant at the time and all were aged between 19 and 36. Six had previous children and five had children who were adopted or being cared for by another family member (Table 2).

**Table 2:** Characteristics of interviewees

**Interviewee**	**status: weeks/gestation (g) or postnatal (p)**	**Age (yrs)**	**use of problem drug (yrs)**	**children (number & age)**
1	23 (g)	29	5	2 : 11 yr, 5 yr
2	12 (p)	27	5	2 : 9 yr, 3 mth
3	11 (p)	19	3	1 : 11 wks
4	10 (p)	23	8	1 : 10 wks
5	20 (p)	19	1.5	1 : 10 wks
6	10 (p)	22	5	1 : 10 wks
7	52 (p)	22	4	2 : 6 yr, 1 yr
8	28 (p)	26	12	1 : 7 mths
9	35 (g)	36	2	0
10	8 (g)	26	5	2 : 10 yr, 4 yr
11	>100 (p)	31	6	2 : 8 yr, 2 yr
12	28 (g)	30	7	2 : 13 yr, 8 yr

Most had a long-term (one year or more) partner at the time of the interview, who was the father of the recent pregnancy. Nearly all interviewees had previously been daily intravenous heroin users and several had also regularly used crack cocaine. All were on a methadone prescription at the time of the interview (variation of 6 – 90 mls daily). Most were stable and successfully reducing but two reported to be also using illicit drugs.

Some pregnancies had not been identified until approximately 22 weeks gestation. The possibility of receiving early prenatal care and support had been reduced in some due to late identification of the pregnancy. The first visit to the Clinic ranged from being the same week up to 12 weeks after first attending General Practice for prenatal care (Table 3).

**Table 3:** Prenatal care of interviewees

**Interviewee**	Identification of pregnancy (wks gestation)***	**1st prenatal appointment (wks gestation)**	**1st Aberdeen Clinic appointment (wks gestation)**
1	8	8	15
2	8	8	20
3	9	10	12
4	6	12	12
5	7	10	10
6	12	12	24
7	20	20	22
8	12	28*	none
9	8	10	14
10	6	6	referred**
11	22	22	22
12	8	8	10

* admitted to maternity hospital by Aberdeen Clinic staff

** awaiting appointment at Aberdeen Clinic at time of interview

*** refers to the moment in the pregnancy the woman realised that she was pregnant.

### Qualitative interviews

#### Referral to the special Clinic

In several cases, referral to the Clinic had been delayed since the family physician/General Practitioner (GP) or the community midwife had either not suggested the Clinic or had not provided appropriate information about the service. Consequently, women did not realise that a high level of multi-agency support was available at the Clinic. Several women only attended the Clinic after they had been admitted to hospital. The midwives in hospital had then referred them:

"I think it was anger. I was five and a half months pregnant. My doctor didn't tell me soon enough really, for me to get right off my methadone."

And

*"I think he *(GP) *could have.... it was when I came here (*hospital) *that they explained it would be better (*to go to the Clinic)."

For some there was a lack of explanation and/or choice:

*"He *(GP) *basically said I had to go there for my treatment."*

This resulted in a feeling of rejection for one woman:

" *It was just like we don't want you up here kind of thing ... passed off because you are an ex junkie."*

And

"I just felt a bit pushed off; I was told they didn't have the services for me .... and I thought well, what do you need to deal with me?"

#### Barriers to accessing care

Most were very wary of going to the Clinic and feared adverse judgements:

"I wasn't very sure ...I thought maybe I would be labelled a junkie. You know you wouldn't be treated the same... at the hospital you are quite anonymous."

And

"I didn't really want to go along. I thought people would look at you and judge you because you were using heroin."

Some avoided the Clinic because they were scared of SW knowing that SW had the power to take a baby away from the mother and into care:

"*I was just too frightened, too scared, thinking they were going to get me into trouble and take her off me the minute she was born."*

Some feared having to go back on methadone either because of its side effects or they felt it was easier to withdraw from heroin, e.g.:

"*I was back using heroin but very little, but I thought it was better using very little than going back on methadone"*

One woman had encountered a very negative attitude from the community midwife and this made her apprehensive to attend the Clinic:

"*You feel really bad because after the first midwife I wasn't too sure what the midwives would be like and whether you could trust them..."*

#### Receiving prenatal care – special Clinic

The overwhelming majority of the women were reassured by the Clinic, they felt in "safe hands" and often expressed more confidence of the treatment there than at their family physician. The Clinic resolved problems where the family physician had been unable to help. Several expressed real enjoyment at going to the Clinic:

"Sometimes I couldn't wait to go because it is like a really familyish atmosphere, they make you feel welcome..."

#### Care provision

Some aspects of the service were important to all interviewees: (a) attitude of staff; (b) consistency of staff; (c) high level of support; (d) reliable information; and (e) integrated care from different services (Table 4). All those interviewed postnatally were positive about the support received and attributed their reduction in drug use to it.

**Table 4:** Themes related to care emerging from interviews

**Theme**	**Example**
Attitude of staff	*"They didn't make you feel like it was something to be ashamed of. They made you feel worth something that you were here, at least you are trying you know..."* *"They had time for me...they wouldn't shove me out the door, they waited till I had finished"*

Continuity of carer	*"... having the same people because they know you and you know them...you don't have to keep repeating yourself, telling your story over and over again."*

High level of support	*"I think it's important to drug users to go more often. I mean if you are going to relapse then there is somebody there to talk to."*

Information	*"They told you all about the drugs. I was quite far on and I didn't know that crack cocaine can kill the baby and I was a regular user. That's extremely important. They don't tell you that anywhere else"*

Integrated care	*"They all interlink with each other, you know....that was really, really handy...it saves you having to travel....it's all in one building, so it was really good."* *"... you don't want to have to keep telling people that you are a user, you only want to say it once for everybody to know."*

"I couldn't see myself stopping without the support, it would have been a disaster."

And

"I think that the Clinic is brilliant and I know a lot of other people that wouldn't have gotten down so far as they have gotten down without the Clinic."

Also

"It was excellent, I don't think I would be clean now without it."

#### Range of care at the special Clinic

The women were asked about the different services they had accessed at the Clinic. Everybody attending the Clinic sees a midwife and a member of staff from the SMS. The use of DA and SW is optional although a woman may be strongly encouraged to talk to the social worker if there are child protection concerns for the baby.

##### (a) Social work

Some women did not want contact with SW because of the stigma involved. Several tried to avoid contact with SW due to previous bad experiences:

" there can be a stigma with them, you know what I mean."

And

"I didn't trust them as a result of that (previous incident). They said it was confidential and turned out it wasn't at all, just made things worse for me."

And

"I didn't want a social worker because I have had them before and I dinnae (=didn't) like their twisting ways they have got. They will tell you one thing but mean another, I dinnae (=didn't) like that. I like somebody to be honest."

Others had been initially reluctant to have contact with SW, but acknowledged that SW had been helpful:

"I was angry for a while, they (SW) sneaked into my life .... but I didn't realise how supportive she would be...I think, now, looking back, I think it was probably the best thing that could have happened because it made me waken up, opened my eyes. I thought this is serious..."

And:

"Excellent help getting a house, she's been excellent really."

##### (b) Voluntary Drugs Agency

Not all of the women had accessed DA. Whilst several relied heavily on the drug worker and valued being able to talk about all their problems, not just those related to the pregnancy. Some felt it was more confidential than with the midwife:

"She doesn't just speak about my drug use, she speaks about everything ...she's really good."

One woman had avoided contact, knowing that the worker was able to supply needles:

"I was scared to talk to her in case I asked her for needles...I just thought if I go speaking to her I might just start asking for needles and then it would make it easier (to relapse)."

#### Receiving prenatal care: hospital

The majority appreciated their hospital care and found the staff friendly and helpful:

"... in hospital there was somebody with me all the time. It's a lot easier to stay off drugs if you're in hospital...the staff were absolutely amazing."

Also:

"They looked after me properly...they took my dinner to me because I couldna (=could not) walk..."

Several appreciated the staff's discreteness regarding their drug use:

"They didn't let anybody else in the ward know why you were there and that you were a drug user...they were discrete..."

Several appreciated the safe environment:

*"You felt sort of safe....you were looked after, it was really nice"*,

An environment that also gave a shelter from home life:

"They took me in for a couple of weeks just to sort of get away from everybody...which worked...I haven't used since then..."

After discharge, the women were able to contact the hospital at any time, if they felt in danger of relapse. Many found this reassuring:

"Any time you feel you are going to use you can always phone them...I haven't actually called them but it's always in my mind that I could."

#### Relationships with professionals

The nature of the relationship between the health care professionals and the women was very variable and depended on both the professional and the client. However, we found several themes.

#### Special clinic

The relationships between the women and the Clinic staff, the midwife and the SMS staff, were reported as very good and friendly. All felt respected and genuinely cared for:

"They're very professional and very friendly."

And

"I felt like I knew them you know.... they were like a little Mother Hubbard, ready to sort you out."

Staff was regarded as supportive even when they saw clients away from the clinic:

"She was really supportive and cuddles and everything. Not like a professional but like a friend, I thought."

And

"They weren't just there for you, they went to see the babies afterwards."

It was reported that the Clinic staff were quite forceful on occasions but this was appreciated, for example:

"They were basically giving me an ultimatum and I had a choice that I had to make and the doctor can't really do that..."

And:

*"The doctor was very understanding, maybe too understanding...it feels good (*now, at the Clinic *) to be pushed in that direction."*

#### Hospital ward

There were positive feelings expressed towards most of the hospital staff, but not to the same extent as at the Clinic. Some were very positive:

"They're very friendly, unbelievably friendly. Non-judgemental and to my partner..."

And

"Oh, they were brilliant ... Some of them was a bit ...stand off, aye, probably because I was on drugs and covered in abscesses ...but some of them was really good."

However, several women trusted and liked them less than the Clinic staff:

"I'm more scared of their reaction on the ward."

And

"Some of them just, they looked at you like shite (=shit)...they just didna (=didn't) want to know you sort of thing, because you're a junkie or whatever."

#### Social work

Generally, women were wary of having any contact with SW but there were examples of valued long-standing relationships. In some cases there was a heavy reliance on the social worker:

"Well I would have probably lost my baby, if it hadn't been for her. She really sort of stuck her neck out for me, because nobody really wanted to give me a chance..."

#### Voluntary Drugs Agency

A few had long-term relationships with a DA worker before falling pregnant. One relationship was obviously close:

"I've been seeing her a long time now, about three years. I like her, yeah. She doesn't just speak about my drug use, she speaks about everything. Any sort of pressures in your life can make you go back using again, she's very aware of that.... she's really good."

One woman had greater trust in her DA worker than her family physician. She had immediately sought advice from her DA worker when she realised about the pregnancy:

"The first thing I did was to phone DA, to tell her I was pregnant...and find out about (effect of) the heroin."

#### Confidentiality

Women are very sensitive to their situation and needed confidentiality. They did not want everyone to know about their drug use and were anxious to hide details about any case conference, a multidisciplinary hearing to further investigate the nature of the drug use and implications for the child. Sometimes the breach of confidentiality had demoralising consequences for the woman drug user:

"Well the woman in the bed next to me, she seemed really nice then she never really spoke to me after the social worker came in and said something about the case conference"

And

"I don't think the social workers give you much privacy. They just came in and started speaking about my case conference in front of like the rest of the ward...."

#### Attitudes

Several themes emerged regarding attitudes to drug use and pregnancy: (a) great anxiety and guilt; and (b) a strong desire to undergo detoxification immediately after discovering the pregnancy.

#### Reaction to pregnancy

The women had experienced great anxiety, guilt and panic:

"It was sheer panic, where do we go, what are the effects on the baby."

And:

"I was really sort of horrified....I honestly thought that if there was a baby inside me there was no possible way that it could be alive."

Many wanted to give up drugs immediately:

"I was using and taking methadone and I thought right, that's it, I've got to stop. But when I stopped using heroin, the methadone wasn't enough and I got very ill."

Some knew that to stop taking drugs could harm the fetus, but still did so:

"When I found out I was pregnant, I know I shouldn't have done it but I stopped taking all the tablets I was on..."

#### Pregnancy as impetus to change

The majority regarded pregnancy as an impetus to change their drug use and their lives. Only those who had suffered from sickness and nausea during their pregnancy did not hold this view:

".... I just thought I felt I couldn't do that, to an unborn child that is so innocent, you're actually giving that baby a habit."

And:

"In a way it was a real opportunity, but I wouldn't say that to other young lassies in case it didn't work like that."

The pregnancy made them see how they had been living before:

"I think when he was born I realised that it's all or nothing – it's all in the past now – it was all shite basically."

And:

"It was when I actually had him that I thought, no, I definitely don't want that life anymore."

The women expressed great determination not to go back to using drugs:

"It dawned on me when he was born; I thought all I have to do is not use. The whole future depends on me not anyone else."

And:

"I don't want to destroy myself on drugs ever again."

The baby had provided a real focus for their lives:

"I know I've wanted to come off drugs for the last four years but there is more of a reason now.... I don't want to go back now."

And:

"I was clean for ten months. As soon as I came back up here I went straight back on it. It was because I never had a goal, but now I have."

The women accepted that it would be more difficult to stay off after the birth, for example:

"It's more of a scarier issue once I have had the baby, because then I'll be on my own. It'll be just me."

In some cases the real focus not to use drugs had been imposed by SW:

"... said they were going to take her into care so that fried my head a bit."

Coming off drugs and having to change friends was an important issue:

"I could count my friends on my hand now whereas before I could have named hundreds of people but now I am very, very cautious about who I choose to be friends with."

#### Guilt

To many it was impossible to face up to being pregnant because they felt so guilty about their drug use and the effect on the baby. For several this encouraged their drug use because it acted as a release:

"I blanked it out.... I didn't want to face up to it...then I got into more drugs to forget it."

And:

"I think it was just so much easier to deny. It was just a bump in my tummy, you couldn't actually see the little person."

#### Failure to identify pregnancy

One woman had not identified her pregnancy until week 22:

"I didn't think that I could fall pregnant while I was on so many drugs. I thought that was impossible."

#### Trends in views

No differences in attitude towards the Clinic or the hospital care could be attributed to the interviewee being either pregnant or postnatal or having positive or negative experiences with the other service providers, DA or SW.

Those who were supported by a partner or parent in addition to clinic care, were more successful in reducing their drug use.

## Discussion

Most women benefited from the multi-disciplinary Clinic as a result of prompt referral from their family physicians, however, for some women an earlier referral would have been an advantage. The majority would have benefited from more information about the Aberdeen Clinic before referral.

All women interviewed preferred the service provided at the Clinic to prenatal care at the GP practice and expressed confidence in the information and treatment provided. Aspects of care which were most highly valued were non-judgemental attitude of staff, reassurance and provision of reliable information, consistency of staff and high level of support in terms of frequency of visit and time given to each client (see Table 4). The women attributed their more stable lifestyle and reduction in drug use almost entirely to the Clinic. The importance of non-judgemental attitudes and consistent, reliable information is recognised [21,37]. The availability of 24-hour support at the hospital was felt to be highly reassuring to clients.

Relationships with professionals varied but were generally very positive with the Clinic staff. The professionals varied individually but also because they were from different agencies. The results indicated the importance of multi-agency care available in a service as a "one-stop shop" as each client had usually developed a close relationship with only one professional. It is reported that staff's attitudes and relationships with clients are often perceived by clients as more important than the quality of the medical care [22,38].

There was strong appreciation when confidentiality was kept and this facilitated the development of trusting relationships between the professionals and clients. Some breaches of confidentiality were noted in this study. Confidentiality is highly important to this client group [39].

Some had tried self-detoxification, not realising the dangers to the baby. However, by contrast, many had tried to block out the pregnancy initially. Their drug use continued and even increased to help to forget everything.

Nearly all thought the pregnancy was a tremendous impetus to change their drug use and their lives and all were determined to stay off illicit drugs and to achieve coming off methadone postnatally. Pregnancy seems to provide a window of opportunity to reduce the drug use. However, there is a difficulty in sustaining a change after the birth and, in this study, those with support from a mother had fared better. Another study highlighted support from a mother or partner as an indicator of good outcomes [40]. There is much emphasis now on child protection [14] and the opportunity to change lifestyle could have important implications for long-term child protection issues.

This study is open to bias, as the women are self-selected due to the nature of the research ethics constraints. Although most clients of the Aberdeen Clinic were on methadone, it is likely that women not coping with their drug use during their pregnancy were less likely to participate in our study. Future studies would benefit from accessing a larger, and, therefore, more representative sample of drug misusing pregnant women. Also drug use is self-reported, which brings with it the possibility of underreporting of illegal drugs in such studies. However, useful findings for service provision as well as ideas for questions to be addressed in a large-scale survey emerged from the data.

## Conclusion

The results of the study highlight the importance for staff who are involved with pregnant women drug users to be aware of the need for sensitivity especially in relation to confidentiality. A breach of confidentiality can be demoralising with possible negative effect on the success of the client in reducing their drug use.

Women drug users commonly experience extreme guilt when they find themselves pregnant and may embark on rapid self-detoxification. There should be more reliable information available for drug-using women on the effects of drug use on the baby as many are unaware or ill informed of the dangers of different types of drugs and the effects of rapid detoxification on the baby.

Pregnancy presents a significant impetus to change lifestyle for the woman drug user and the high level multidisciplinary support provided was a clear aid to achieving such change. Multi-agency integrated care in the prenatal period has been shown to be preferred by this client group to care by the local family physician. It appears that the combination of different aspects of health and social care of the multi-agency clinic (one-stop shop) was contributory to the success of the clients in reducing drug use and stabilising lifestyles, as reported by the interviewees. It has been recognised that properly coordinated services help avoid providing a confusing array of services and appointments for pregnant drug users [41]. In order that this multi-agency approach may work successfully, family physicians should be better informed about it, refer early and provide relevant information to their clients.

Further research is required to establish whether the support provided by the Clinic produced sustained changes in lifestyle. As previously suggested we also need more research into the long-term outcomes for the child [42].

The risk to women drug users and their children is an increasing public health problem, which is in line with the increasing use of drugs in society. A greater awareness of the needs and feelings of the pregnant drug user can allow more effective prevention strategies to be implemented.

## Competing interests

The author(s) declare that they have no competing interests.

## Authors' contributions

JH carried out the data collection and analysis for this study as part of her M.Sc. in Health Services and Public Health Research. EvT supervised this research project and participated at all stages of the study. Both authors have written several drafts and approved the final manuscript.

## Pre-publication history

The pre-publication history for this paper can be accessed here:


